# OFF bipolar cells in macaque retina: type-specific connectivity in the outer and inner synaptic layers

**DOI:** 10.3389/fnana.2015.00122

**Published:** 2015-10-06

**Authors:** Yoshihiko Tsukamoto, Naoko Omi

**Affiliations:** ^1^Studio Retina, SatonakaNishinomiya, Japan; ^2^Department of Biology, Hyogo College of MedicineNishinomiya, Japan

**Keywords:** monkey retina, basal synapse, ribbon synapse, glutamate receptor, ganglion cell, serial section electron microscopy, network, vision

## Abstract

OFF bipolar cells in the macaque retina were recently classified into five types: flat midget bipolar (FMB) and diffuse bipolar (DB) 1, 2, 3a, and 3b. We examined all parallel pathways from cone photoreceptors via OFF bipolar cells to parasol and midget ganglion cells by serial section transmission electron microscopy. Basal contacts of OFF bipolar cells to cone pedicles were previously categorized as triad-associated (TA) and non-TA (NTA). The latter was further divided into two groups located in the middle and marginal areas of the pedicle at the present eccentricity of 15°. We then mapped the distributions of all three basal contacts of the five OFF bipolar cell types in the same area of cone pedicles. TA contacts were more numerous than NTA contacts in FMB (93%), DB1 (67%), and DB3a (81%) cells, but less in DB2 (30%) and DB3b (21%) cells. Cluster analysis of these contact parameters reconfirmed five distinct OFF bipolar cell types and showed these positional configurations of basal synapses to be cell type-specific. This architecture is thought to provide a spatial framework for the interstitial diffusion and local uptake of the neurotransmitter (glutamate) that spills over from ribbon synapses. All five OFF bipolar cell types formed ribbon-synaptic contacts to both parasol and midget ganglion cells. DB2 and 3a, DB1 and 3b, and FMB predominantly, moderately, and negligibly contacted parasol ganglion cells, respectively. FMB almost exclusively contacted midget ganglion cells, to which DB1 provided dominant output (58%), and DB2, 3a, and 3b provided between 3% and 10% of their output. Consequently, the cone signal sampling routes of a midget ganglion cell consisted of two substructures: the narrow (mainly 2-3 cones) FMB pathway and the wide (mainly 10 cones) DB pathway, where connection strength was four-fold greater in the FMB than DB pathway. The narrow and strong FMB pathway may confer the highest spatial resolution and sporadically may include blue cone signals.

## Introduction

The parallel analysis of visual images begins in the retina distal to ganglion cells; different types of bipolar cells divergently and convergently transmit photoreceptor signals to ganglion cells (Dowling and Boycott, 1966; Boycott and Dowling, 1969; Kolb, 1970; Masland, 2001; Wässle, 2004; Field and Chichilnisky, 2007; Euler et al., 2014). Macaque OFF(-center) bipolar cells were first classified into four types by Boycott and Wässle (1991) using the Golgi staining method. Recently, Puthussery et al. (2013) have immunochemically stained the newly defined diffuse bipolar (DB) 3b type. They recorded its electrophysiological connectivity with parasol ganglion cells; however, whether these cells contribute to the parasol ganglion/magnocellular pathway is yet unclear. Tsukamoto and Omi (2013, 2014) have independently shown that DB3b cells have novel connections with rod photoreceptors and divided all OFF bipolar cells into five types, flat midget bipolar (FMB), DB1, 2, 3a, and 3b, based on the quantitative morphology of their axon terminals. Since the classification of OFF bipolar cells has been established, it is possible to define the parallel pathways of each via the basal synapses between cone pedicles and bipolar dendrites and the ribbon synapses between bipolar axons and ganglion cell dendrites.

Boycott and Hopkins (1993) have divided the basal synapses into triad-associated (TA) and non-TA (NTA) groups. We adopted this scheme for the present analysis. However, on analysis, we recognized the necessity to further divide NTA basal synapses into two subgroups, middle and marginal NTA basal synapses, according to their distances from the nearest membrane invagination. The association between the structural architecture of basal synapses and the postsynaptic responses of OFF bipolar cells was explored by electrophysiological experiments in ground squirrel retinas (DeVries et al., 2006; Szmajda and DeVries, 2011). One of the key findings was that the neurotransmitter glutamate could spill over from the ribbon synapse-associated invaginations and effectively reach postsynaptic receptors at remote basal contacts, depending on the diffusion constant and the local uptake efficiency. Our study attempts to clarify the structural framework of this remote chemical transmission along macaque cone pedicles. This first stage of parallel processing is further relayed to the output channels of ganglion cells.

Two contrasting functional channels have been defined in the lateral geniculate nucleus (LGN) of primates (Derrington and Lennie, 1984; Kaplan and Shapley, 1986; Watanabe and Rodieck, 1989; Schiller et al., 1990). They are the magnocellular channel that responds transiently with high contrast sensitivity via parasol ganglion cells and the parvocellular channel that responds steadily with low contrast sensitivity via midget ganglion cells. The highest spatial resolution and smallest receptive field center sizes were found to be carried by parvocellular neurons at any eccentricity. However, the overall receptive field center parameters of parvocellular cells overlapped greatly with those of magnocellular cells with only subtle quantitative differences (Derrington and Lennie, 1984; Spear et al., 1994; Levitt et al., 2001). This perplexing dichotomy may originate in neural connectivity in both retina and LGN. Here we focused on the precise connectivity within the retina.

In this study, we first characterized the basal synapses of OFF bipolar cell dendrites with cone pedicles. Second, we performed cluster analysis of OFF bipolar cells using four variables concerning TA and NTA contacts. Third, we examined all types of bipolar cell transmission routes from cones to parasol and midget ganglion cells. Fourth, we analyzed the cone sampling routes of a midget ganglion cell based on the connectivity strength as evaluated by the product of the synaptic contact numbers at cone-bipolar and bipolar-ganglion interfaces.

## Materials and methods

### Serial section transmission electron microscopy

#### Macaque monkey retina

A series of 817 radial sections, each 90 nm in thickness (73.5 μm total thickness), was prepared for serial section transmission electron microscopy from the posterior right retina of a 7-year-old female Japanese monkey (*Macaca fuscata*, 6.5 kg). This animal was provided for our study by the psychophysical research group in the (former) Electrotechnical Laboratory in Ministry of International Trade and Industry. The series is the same as previously used by Tsukamoto and Omi (2014) in which the procedure of sample preparation was described in detail. The procedure was performed in compliance with the Guide for the Care and Use of Experimental Animals (Hyogo College of Medicine).

Here, we briefly describe the electron-density staining necessary to analyze the different cone-bipolar-ganglion cell pathways. After dual perfusion with aldehyde fixative via intraocular and intravascular passages, tissue blocks of retina with intact sclera and choroid were isolated, post-fixed with a mixture of 2% osmium tetroxide and 1% potassium ferricyanide, and stained *en bloc* with 3% uranyl acetate in 80% methanol. The metal ions contained in these solutions provided some degree of density contrast to visualize subcellular components. Blocks were embedded in Araldite resin and cut in serial sections. Sections were mounted on 120 formvar-coated single-slot grids and stained with 3% uranyl acetate in 80% methanol and Reynolds' lead citrate. These final stains provided sufficient image contrast to discriminate fine cytological features.

Electron micrographs of the section series were acquired at both 400 × and 3000 × using a JEM 1220 electron microscope (Jeol Ltd, Tokyo, Japan) at the Joint-Use Research Facilities of Hyogo College of Medicine. Twenty-four overlapping negative images were acquired from each individual section at 3000 × to capture a 90 μm × 187 μm area covering the outer plexiform layer (OPL) to ganglion cell layer in a 4 × 6 montage. These images were enlarged four-fold; thus, the final magnification of prints used for image analysis was 12000 ×.

#### Examination area

The examination area was located 3.00–3.25 mm temporal to the foveal center and its center was approximately 15° from the foveal center. The densities of rod spherules, cone pedicles, and ganglion cells in this region were 172 × 10^3^ spherules/mm^2^, 12.6 × 10^3^ pedicles/mm^2^, and 11.3 × 10^3^ cells/mm^2^. The cone pedicles were approximately 45 μm far from the cone cell bodies in planar distance via Henle's fibers. Inner and outer segments of the cones protruded perpendicularly upward from the cell bodies to the retinal surface. The density of cone cell bodies was approximately equal to that of cone pedicles in this eccentricity. The spherule to pedicle ratio was 13.6: 1 and the pedicle to ganglion ratio was 1.1: 1. The specimens of retina along the horizontal meridian were cut together with the choroid and sclera to protect the retina from planar shrinkage (Tsukamoto et al., 1992); therefore, no shrinkage correction was undertaken.

Several previous studies reported that the area with highest rod density was located along the superior vertical meridian in both macaque retina (177 × 10^3^ rods/mm^2^; Packer et al., 1989) and human retina (158-189 × 10^3^ rods/mm^2^; Curcio and Allen, 1990); however, the peak rod density along the temporal horizontal meridian was as high as 160 × 10^3^ rods/mm^2^ (Mariani et al., 1984) or 120 × 10^3^ rods/mm^2^ (Adams et al., 1974; Packer et al., 1989). Thus, the retinal locus we examined was regarded as the peak rod density area along the horizontal meridian. A similar area at 3 mm eccentricity in the temporal retina of *M. fascicularis* has been investigated by Wässle et al. (1989, 1990). They showed that the cone to ganglion ratio was approximately 1 : 1, which is almost equal to 1.1 : 1 of our sample. This cone to ganglion ratio is far less than necessary for foveal circuitry, where one cone requires more than two ganglion cells, ON and OFF midget ganglion cells. Thus, our present examination area is characterized by high-rod density and the features of peripheral circuits.

### Data analysis

#### Classification of short- and middle/long- wavelength sensitive cones

Short-wavelength-sensitive (S-) cones can be identified by the innervation of the invaginating dendrites of blue bipolar cells (Mariani, 1984; Kouyama and Marshak, 1992; Wässle et al., 1994). In this study (data not shown), we found 18 blue bipolar cells connected to two small bistratified ganglion cells assumed to receive ON blue signals (Dacey and Lee, 1994; Calkins et al., 1998; Dacey et al., 2014). These blue bipolar cells had more than one synaptic contact with any one of the 10 cone pedicles that we classified as S-cones. The remaining cones studied were classified as middle/long-wavelength sensitive (M/L-) cones. Further, we reconfirmed that the S-cone pedicles were distinctly smaller in area and volume than the M/L-cone pedicles according to the anatomical criteria described by Kolb (1991).

#### Statistics and cone-ganglion connection strength

Quantitative data are usually presented as the mean ± standard deviation (SD), number of samples (n) in tables and figures. When sample numbers were very small, only mean values are presented. Cluster analysis (Ward's joining method) was applied to OFF bipolar cells using Statistica 06J (Statsoft Japan, Tokyo, Japan).

The number of synaptic contacts is an essential quantitative parameter to assess the efficacy of synaptic transmission under the assumption that individual contacts are equivalent, although we require knowledge regarding various other aspects to completely understand the synaptic dynamics. In this analysis, we focused on the cone–OFF bipolar–midget ganglion synaptic pathways. We evaluated circuital connection strength by the product of the synaptic contact numbers at two interfaces between cones and OFF bipolar cells and between OFF bipolar and midget ganglion cells as in the following formula. Each ganglion cell connects to several bipolar cells via variable numbers of synaptic contacts, where w_*gi*_ is the number of contacts between the *g*-th ganglion and *i*-th bipolar cell (*i* = *1, 2*, ⋯ *, m*). In turn, each bipolar cell connects to several cones via variable numbers of synaptic contacts, where *v*_*ij*_ is the number of contacts between the *i*-th bipolar and *j*-th cone (*j* = *1, 2*, ⋯ *, n*). The sum of the products of contact numbers w_*gi*_ *v*_*ij*_ for all *m* bipolar cells gives the connection strength between the *g*-th ganglion cell and *j*-th cone.

$$p_{gj}=\sum_{i\;=\;1}^{m}w_{\text{gi}}v_{\text{ij}}=w_{\text{g}1}v_{1\text{j}}+w_{\text{g}2}v_{2\text{j}}+\cdots +w_{\text{gm}}v_{\text{mj}}$$

The sum of these contact number products for all convergent cones yields an estimate of the total connection strength (*P*_*g*_) of the *g*-th ganglion cell with its cone field:

$$P_{\text{g}}=\sum_{j\;=\;1}^{\text{n}}p_{gj}=p_{g1}+p_{g2}+\cdots +p_{gn}.$$

This formulation is widely used for the computational analysis of three-layer networks (Jordan, 1986). The retinal cone–bipolar–ganglion circuitry is a representative real three-layer neural system. The *p*_*gj*_ may be similar in concept to the sampling strength (*a*_*gj*_) of the *j*-th cone by the *g*-th receptive field used by Field et al. (2010). The precisely and extensively reconstructed retinal circuits of both cone-bipolar and bipolar–ganglion interfaces are required for research from the computational neuroscience perspective. In this study, we count individual synaptic contacts of bipolar cells with both cones and ganglion cells.

## Results

### Positional configurations of basal contacts

**Figure 1:**
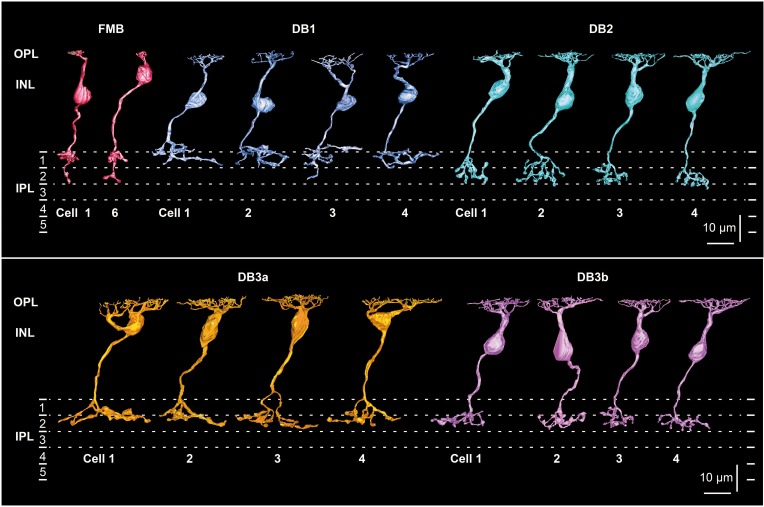
**Morphology and stratification of FMB, DB1, DB2, DB3a, and DB3b types of OFF bipolar cells**. FMB cell-1 and cell-6 (FMB-1 and FMB-6) are connected to M/L and S cones, respectively. Four cells (1-4) are displayed for each DB type. Each stratum of the IPL (1-5) is 6 μm thick. Strata 1-2 comprise the OFF sublamina.

To characterize the five types of OFF bipolar cell in terms of the synaptic connectivity with cones and parasol and midget ganglion cells, we fully reconstructed six cells of FMB type and four cells of each DB type. FMB cells were divided into two groups according to the cone type contacted, M/L or S. We used M/L-cone-connected FMB cells as the representative type for quantitative comparison to DB cells. Figure 1 shows an example of one FMB cell (cell 1) connected to an M/L-cone and another (cell 6) connected to an S-cone. A tiny process, termed “axon tail” (Jusuf et al., 2006), was almost always observed that extended downward from the axon terminal bulb of FMB cells. Four cells (1-4) of each DB type (1, 2, 3a, and 3b) are shown in Figure 1. The depth of the DB cell axon terminal in the inner plexiform layer (IPL) descended in the order DB1, 3a, 3b, and 2. When more than four cells were required for characterizing the connectivity of DB cell dendrites with cones, we added the reconstruction of the dendritic portion of a few more cells for each type in the following analyses.

#### Tiling cones with different dendritic overlaps

**Figure 2:**
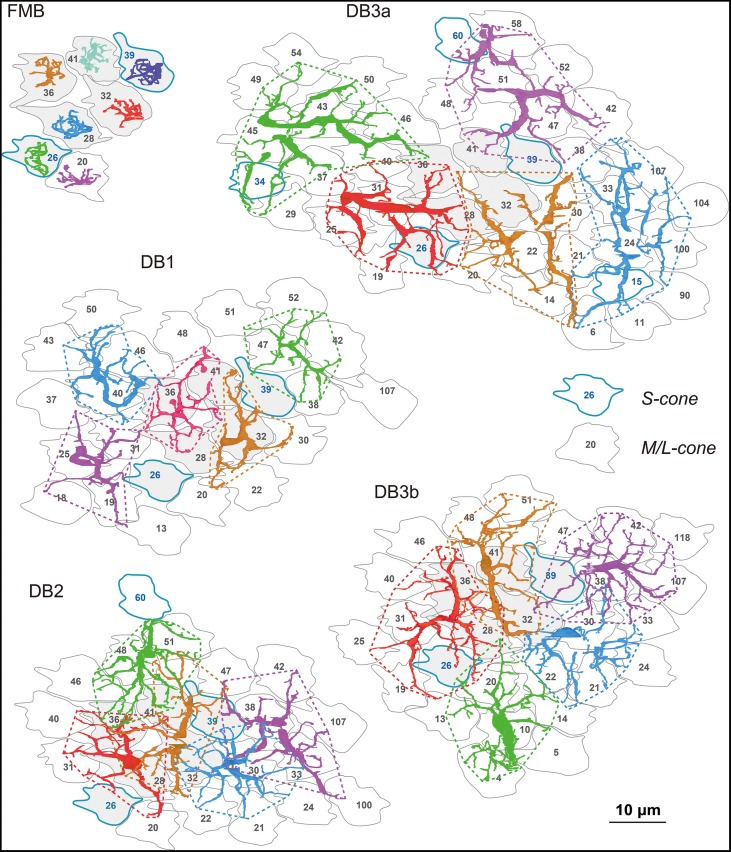
**Dendrites of each OFF bipolar cell type covering arrays of cone photoreceptor pedicles**. The dendrites of seven (FMB) or five (DB) cells in each subgroup are shown in different colors for clarity. Cone pedicles are labeled by serial numbers and accordingly in the following figures. M/L-cones are labeled with black letters and S cones with blue letters and contours. Each type tiles an area of cone pedicles and most of each cone area is innervated by the dendrites of the five types of OFF bipolar cells. Six central pedicles (26, 28, 32, 36, 39, and 41) are designated in gray for reference.

The OFF bipolar dendritic fields of each type tiled the same field of cone pedicles (Figure 2) with the different degrees of overlapping. The dendritic field of every FMB cell examined innervated only one cone. The average number of cones converging onto a DB cell with at least one synaptic contact was approximately 7 for DB1, 8 for DB2, 9 for DB3b, and 10 for DB3a, indicative of increasing dendritic field size in the rank order DB1, 2, 3b, and 3a. Thus, a DB2 cell collected more cones than a DB1 cell, but the total number of cones converging onto five DB2 cells was 23 compared with 26 for five DB1 cells. The reason for this reversal was that the dendrites of DB2 cells overlapped more than those of DB1 cells, indicative of the shorter sampling period of DB2. The dendritic fields showed almost no overlapping among DB1 cells or DB3a cells. The dendritic fields of DB3b were slightly overlapping. However, a survey at this level of resolution is insufficient to reveal individual synaptic contacts, which are examined next.

#### Triad-associated (TA), and middle and marginal non-triad-associated (NTA) basal synapses

At 3 mm eccentricity of our sample series, the diameter of the pedicle base excluding the marginal extensions was approximately 8 μm for M/L-cones and 6–7 μm for S-cones (Kolb, 1991). Nevertheless, the number of ribbons per pedicle was 31.3 ± 3.4 (mean ± SD) for M/L-cones (*n* = 23) and 31.8 ± 2.8 for S-cones (*n* = 10). No significant difference in ribbon number was observed between M/L- and S-cones. It is known (Chun et al., 1996; Haverkamp et al., 2001b) that the average number of ribbons varies depending on eccentricity between 20 in the central retina and 50 in the far-peripheral retina. The present value of 31 indicates the property of cone pedicles in the mid-peripheral retina. In a few cases per pedicle, two separate invaginations were situated under a single large ribbon; therefore, the number of invaginations may be slightly larger than the number of ribbons.

**Figure 3:**
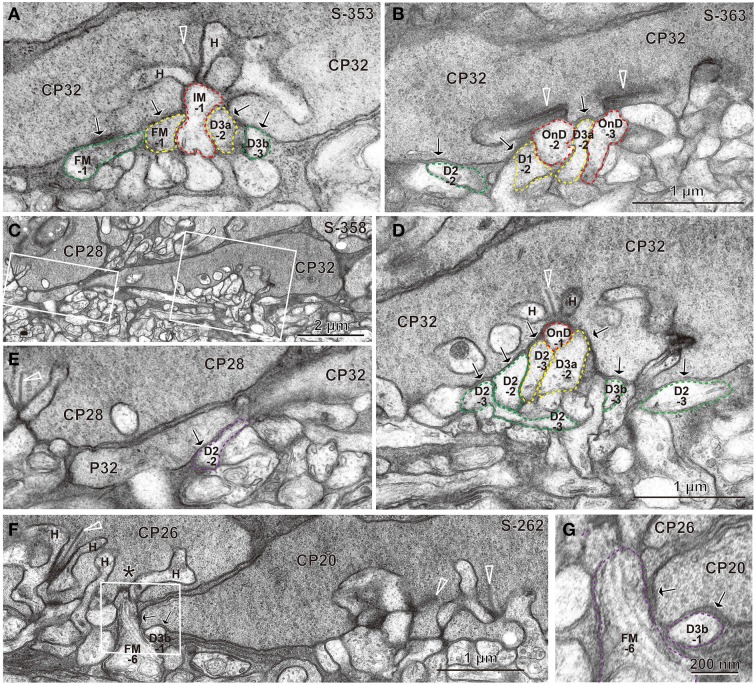
**Electron micrographs of synapses between bipolar cell dendrites and cone pedicles (CP). (A)** An invaginating synapse (IMB-1 in red) of an ON MB cell under the ribbon (arrowhead) flanked by two horizontal processes (H), “TA” synapses of cells FMB-1 and DB3a-2 (in yellow), and two “middle NTA” synapses of cells FMB-1 and DB3b-3 (in green) are demarcated. **(B)** ON DB cells form two invaginating synapses (OnD-2 and OnD-3 in red), adjacent to which are two “TA” synapses of cells DB1-2 and DB3a-2 (in yellow). Nearby and to the left is one “middle NTA” synapse of cell DB2-2 (in green). **(C)** Two rectangular areas are enlarged in **(D,E)**. **(D)** An ON DB cell (OnD-1 in red contour) projects an invaginating dendrite immediately underneath a ribbon, just adjacent to which two dendrites (of cells DB2-3 and 3a-2 in yellow) form “TA” synapses. Four dendrites (of cells DB2-3, 2-2, 3b-3, and 2-3 in green) form “middle NTA” synapses, which are separated by two or more other processes from the ribbon but are still less than 1.5 μm from it. **(E)** A dendrite of cell DB2-2 (in violet) forms one “marginal NTA” synaptic contact with the marginal extension of CP32. This contact is located about 3.5 μm from the nearest ribbon of CP32 but only about 2 μm from the nearest ribbon of CP28 (arrowhead). **(F)** The marginal extension of CP20 is located under CP26, where two basal contacts with CP20 are much closer to the nearest ribbon area (^*^) of CP26 than that of CP20. The rectangular area is enlarged in **(G)**. **(G)** “Marginal NTA” synapse of cells FMB-6 and DB3b-1 with CP20. S-353 stands for section 353 etc. Bipolar letterings are abbreviated without “B.”

Under each ribbon, as postsynaptic dendrites, existed the triad that consisted of one ON bipolar invaginating dendrite and two horizontal processes. In the basal surface, there were basal synaptic contacts created by OFF bipolar cell dendrites. They were categorized into three classes depending on their varying distances from the triad. In accordance with previous studies (Boycott and Hopkins, 1993; Hopkins and Boycott, 1995, 1996, 1997; Calkins et al., 1996), we first distinguished TA and NTA basal synapses at cone pedicles as shown in the electron micrographs of Figure 3.

A typical triad consisting of an invaginating dendrite (IMB-1, contoured by red) of the ON midget bipolar (IMB) cell in front of a ribbon and two horizontal cell processes flanking the ribbon is shown in Figure 3A. The junctions between the pedicle base and the OFF bipolar dendrites (FMB-1 and DB3a-2, contoured by yellow) adjacent to the fully invaginating dendrite (IMB-1) were classified as TA basal synapses. Alternatively, the junctions between the pedicle base and the OFF bipolar dendrites (FMB-1 and DB3b-3, contoured by green) separated from the ribbon zone by two intervening processes were classified as NTA basal synapses. Similar examples are found in Figure 3B, where two ribbons were cut longitudinally. Adjacent to two fully invaginating dendrites (cells ON DB-2 and -3) are seen two TA synapses of cells DB1-2 and DB3a-2, apart from which is a NTA synapse of DB2-2.

During this inspection, we found basal synaptic contacts located at the marginal area of the pedicle base or at the filopodial processes extending from the pedicle margin. For example, as shown in Figures 3C–E, two TA synapses (cells DB2-3 and DB3a-2) and four NTA synapses (cells DB2-2, DB2-3, DB2-3, and DB3b-3) were formed in the central area of the pedicle base (CP32) while another NTA synapse (cell DB2-2) was observed at the marginal area of the same pedicle. The nearest ribbon zone to this marginal NTA synapse between CP32 and DB2-2 was not that contacting pedicle CP32 (3.5 μm distant) but rather the ribbon zone in the adjacent pedicle CP28 (about 2 μm away). In another case, a marginal NTA (cell DB3b-1) was much closer to the nearest ribbon zone (<1 μm) of the adjacent pedicle (CP26) than to the ribbon zone (approximately 3 μm) of the contacting pedicle (CP20) (Figures 3F,G).

We defined a “TA” basal synapse as a contact between the pedicle base and OFF bipolar dendrite that was adjacent to a fully invaginating dendrite, a “middle NTA” basal synapse as an OFF bipolar-pedicle contact that was separated by two or more other processes but located less than 1.5 μm along the basal line from the ribbon zone, and a “marginal NTA” basal synapse as an OFF bipolar-pedicle contact located more than 1.5 μm along the basal line from the nearest ribbon zone of the contacting pedicle. The depth of a membrane invagination was roughly 0.5 μm; therefore, a border of 1.5 μm implies a circular area of radius 1 μm from the invagination. As the area of diameter 8 μm is 50 μm^2^ for the M/L-cone pedicle base, a ribbon occupied 1.6 μm^2^ on average, which corresponds to a radius of 0.7 μm. Based on these conditions, all NTA synapses within the middle area of the pedicle base were classified as “middle NTA synapses” and other NTA synapses in the marginal area and filopodial processes of the pedicle base as “marginal NTA synapses.”

#### Distribution of the three classes of basal contacts

**Figure 4:**
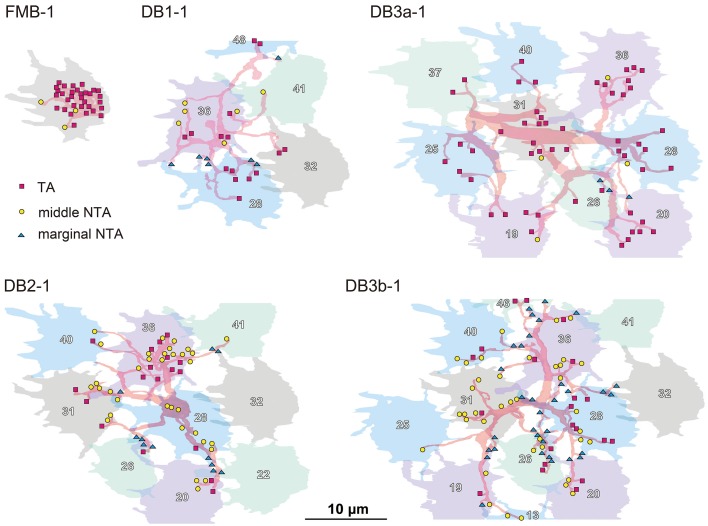
**Distributions of the three positional classes of basal synapses for the five OFF bipolar cell types**. The dendrites (pale red) of the five types of OFF bipolar cells form basal synapses with cone pedicles (different colors with serial pedicle numbers). The synaptic contacts are categorized according to their basal positions as TA (magenta square), middle NTA (yellow circle), and marginal NTA (cyan triangle).

The distribution of the three classes of basal synapses are pictorially displayed for each of the five OFF bipolar cell types in Figure 4. For FMB cells, the majority of basal synapses had TA contacts. Among DB types, TA contacts were most abundant in DB3a cells, followed by DB1 cells. In contrast, middle NTA contacts constituted nearly half the basal synapses of DB2 and DB3b cells. Both TA and middle NTA contacts lay in the central area of the pedicle base. The marginal area and filopodial extensions of the pedicle base were mostly innervated by marginal NTA contacts. Such contacts were absent in FMB cells but present in DB cells at an increasing frequency in the rank order of DB3a (least), DB1, DB2, and DB3b (most).

**Figure 5:**
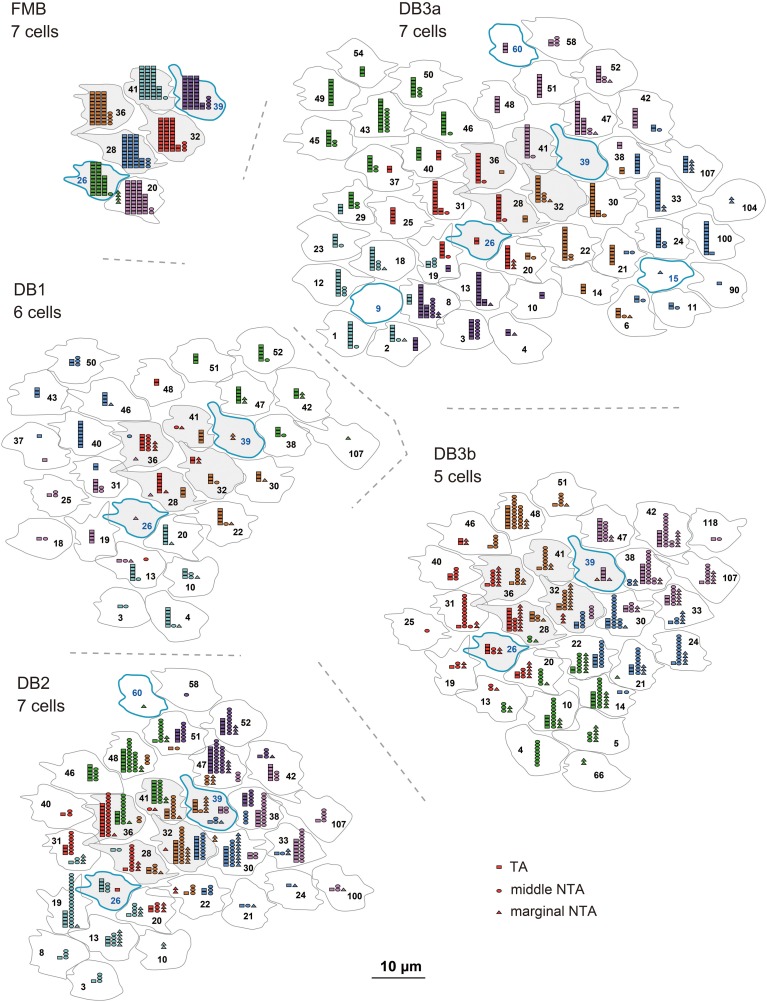
**Abundance of each basal synapse class over the entire cone area for each OFF bipolar cell type**. A group of OFF bipolar cells of the same type form basal synapses (TA in rectangles, middle NTA in ellipses, and marginal NTA in triangles) with a layer of cone pedicles. The first five members of each DB type are designated by the same colors as in Figure 2. Six central pedicles (26, 28, 32, 36, 39, and 41) are designated in gray for reference.

In Figure 5, the quantitative profiles of these three basal synapse classes are illustrated for 5-7 contiguous cells of each bipolar cell type and the corresponding field of many cones (7 cones for FMB and 29-51 cones for DB). Every cone connected to only one FMB cell, whereas 5–9 cones connected to each DB1, 6-10 to each DB2, and 8-12 cones to each DB3a and DB3b cell. Several cones in the middle region (designated in gray) of each field were fully innervated by the dendrites of each DB type, which allowed for an analysis of cone divergence. A cone had contacts only with a single DB1 or DB3a cell nearby. In contrast, a cone diverged to two DB2 or DB3b cells in the neighborhood. These differences in divergence correlated with the aforementioned degree of overlapping dendrites (Figure 2).

### Statistics of basal contacts and cluster analysis

**Figure 6:**
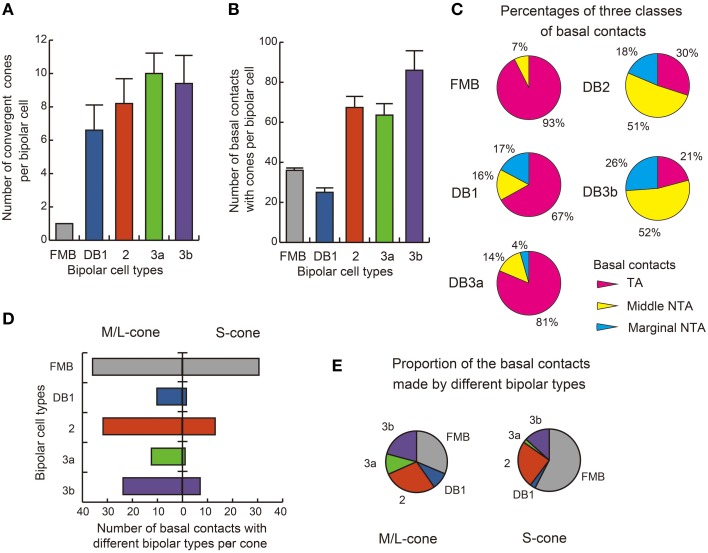
**Summary of basal synapse counts**. **(A)** The number of cones converging onto each bipolar cell (every cone was equally counted as unity regardless of the number of synaptic contacts). **(B)** The total number of contacts of the three positional classes between bipolar dendrites and cone pedicles. The data are averaged for each OFF bipolar cell type (*n* = 5) with SD **(A,B). (C)** Pie charts showing the average proportions of the three positional classes of basal synapses. All FMB cells (*n* = 5) are of the M/L cone-connected type. **(D)** Comparison of S cone (right-handed bars, *n* = 2) and M/L cone (left-handed bars, *n* = 4) divergence for the five types of bipolar cells. The abscissa is the average number of basal contacts of a cone with each type of bipolar cell. **(E)** Pie charts showing comparison of S-cones and M/L-cones in the innervation of different bipolar types.

The distribution of basal synapse classes varied markedly among OFF bipolar cell types (Table 1, Figures 6A,B). The proportion of TA contacts decreased substantially from 93% for FMB to 21% for DB3b (rank order FMB, DB3a, 1, 2, and 3b) and, conversely, that of NTA contacts increased in this same rank order. The proportions of middle NTA contacts were 7% for FMB, 14%-17% for DB3a and DB1, and 51%-52% for DB2 and DB3b, while the proportions of marginal NTA contacts were none for FMB, 4% for DB3a, 17%-18% for DB1 and DB2, and 26% for DB3b (Figure 6C).

**Table 1:** **Positional configurations of the basal synaptic contacts with cone pedicles for the five types of OFF bipolar cells**.

**(A) The present study**^**a**^							**(B) Other researcher's study**		
**Bipolar cell type**	**TA**	**NTA**	**Total**	**TA**	**NTA**		**Bipolar cell type**	**TA**	**NTA**
	**Mean ± SD**	**Mean ± SD**	**Mean ± SD**	**%**	**%**			**%**	**%**
FMB	33.0 ± 2.2	2.6 ± 1.1	35.6 ± 1.5	93	7		FMB^b^	79	21
DB1	16.8 ± 3.3	8.2 ± 3.3	25.0 ± 2.2	67	33		DB1^b^	59	41
DB2	20.4 ± 2.9	47.0 ± 3.9	67.4 ± 5.6	30	70		DB2^b^	50	50
DB3a	51.6 ± 4.0	12.0 ± 4.4	63.6 ± 5.7	81	19		DB2^c^	43	57
DB3b	18.4 ± 6.9	67.6 ± 4.7	86.0 ± 9.7	21	79		DB3^b^	75	25

a *Number of samples: n = 5 for all cases. M/L-cone-connected FMB cells were chosen for comparison with DB cells*.

b *Data of macaque monkey (Macaca mulatta) (Hopkins and Boycott, 1997)*.

c *Data of velvet monkey (Boycott and Hopkins, 1993)*.

We found substantial differences in the number of basal contacts from M/L- and S-cones among DB cell types (Table 2). In our sample, two S-cones (CP26 and 39) were fully innervated by the dendrites of all DB types, allowing an analysis of their synaptic partners. Likewise, four M/L-cones (CP28, 32, 36, and 41) were fully innervated by the dendrites of all DB types. The comparison between S-cone and M/L-cone divergence to all OFF bipolar cell types via basal contacts is displayed in Figures 6D,E. Generally, S-cones formed fewer basal contacts with DB cells than M/L-cones. This was consistent with the previous finding by Lee and Grünert (2007) that connections of DB cells were biased against S-cones in primate retina. More specifically in Figure 5, DB1 and DB3a cells had fewer basal contacts with S-cones compared to DB2 and DB3b cells. For example, a DB1 cell had only one marginal NTA contact with CP26 (S). The dendritic tip at this marginal NTA contact was very close to the adjacent CP19 (M/L). Consequently the receptor on this tip may receive more spillover glutamate from CP19 than CP26. CP39 (S) eventually had no contacts with DB3a cells. Furthermore, it was noted on comparison with TA contents (Figure 6C) that these DB1 and DB3a types had the higher proportion of TA basal contacts (67 and 81% respectively) than DB2 and DB3b.

**Table 2:** **Convergence of cones onto OFF bipolar cell types and the number of basal contacts for divergence of M/L- and S-cones to OFF bipolar cell types**.

**Bipolar cell type**	**Cones converging onto a bipolar cell**	**Basal contacts per M/L cone**		**Basal contacts per S cone**	
	**Mean ± SD**	**Mean**	**%**	**Mean**	**%**
FMB	1.0 ± 0.0	35.8	31.6	30.5	57.6
DB1	6.6 ± 1.5	10	8.9	1.5	2.8
DB2	8.2 ± 1.5	31.5	27.9	13	24.5
DB3a	10.0 ± 1.2	12.3	10.8	1	1.9
DB3b	9.4 ± 1.7	23.5	20.8	7	13.2
	Total	113	100	53	100

n = 5 for the number of convergent cones, n = 4 for the mean of M/L cones, and n = 2 for the mean of S cones.

**Figure 7:**
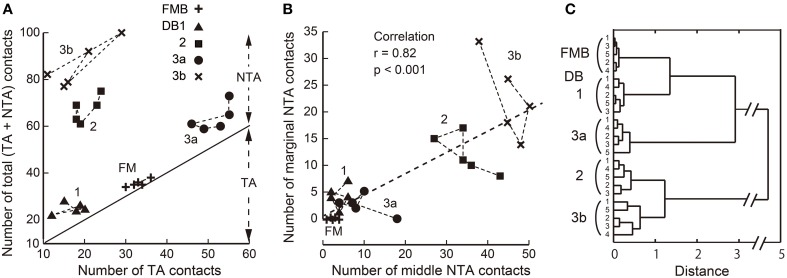
**Scatter plots of TA, middle NTA, and marginal NTA basal contacts and their clustering among the five OFF bipolar cell types**. **(A)** Relationship between the total number of basal contacts and the number of TA contacts per cell. The slope line indicates the TA component of a total (TA + NTA) along the ordinate. **(B)** Numbers of marginal and middle NTA contacts are strongly correlated for the entire OFF bipolar cell population. **(C)** Dendrogram of cluster analysis (Ward's method) of 25 OFF bipolar cells using the four variables plotted in **(A,B)**. Five clusters are differentiated.

In summary, the number of basal contacts and the proportions of the three basal contact classes appear distinct for each OFF bipolar cell type. Indeed, the scatter plot of the total number of contacts for each type vs. the number of TA contacts for each type (Figure 7A) demonstrates this clustering within types and the separation between types. The scatter plot of the middle and marginal NTA contacts (Figure 7B) reveals a significant correlation for DB2 and DB3b cells, indicating a tendency for these contacts to be concomitantly expressed.

We applied cluster analysis to the 25 OFF bipolar cells described (Figure 7C) using four variables: the individual total numbers of TA, middle NTA, and marginal NTA contacts and the total number of basal contacts normalized to the maximal value. This analysis reconfirmed the validity of our classification of the 25 cells into five types and demonstrated that these positional differences of OFF bipolar basal synapses were type-specific characteristics.

### Connections to parasol and midget ganglion cells

**Figure 8:**
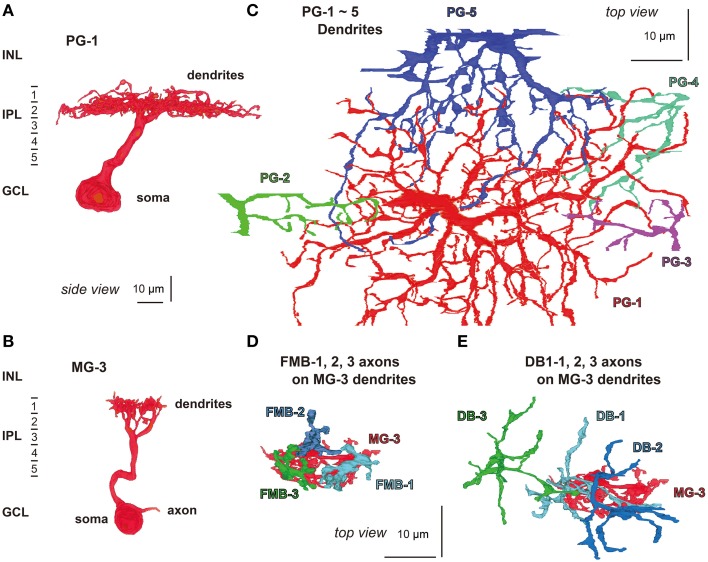
**Morphology and stratification of parasol (PG-1) and midget (MG-3) ganglion cells**. **(A)** A parasol ganglion cell (PG-1) and **(B)** a midget ganglion cell (MG-3) in side view. **(C)** The dendrites of a parasol ganglion cell (PG-1: red) and four others (PG-2 to -5: green, blue-violet, blue-green, and red-violet, respectively) in top view. **(D,E)** Cell MG-3 dendrites (red) are innervated by the axon terminals of three FMB cells (FMB-1, -2, and -3: light blue, dark blue, and green, respectively) and three DB1 cells (DB1-1, -2, and -3: in the same colors) in top view.

We attempted to obtain the quantitative profile of five types of bipolar cell in terms of the output ribbon synapses directed to parasol and midget ganglion cells. A total of 20 OFF bipolar cells, four cells for each type, were sampled from the middle examination area. The dendrites of five parasol (PG-1 to -5) and 15 midget ganglion (MG-1 to -15) cells were identified to be the postsynaptic cells to receive all synaptic contacts provided by 20 bipolar cells. The dendrites of a parasol ganglion cell (PG-1) were mainly distributed in stratum 2 of the IPL (Figure 8A), where the axon terminals of DB2, 3a, and 3b cells were also located (Figure 1). Likewise, the dendrites of a midget ganglion cell (MG-3) were in stratum 1 (Figure 8B), in which the axon terminals of FMB and DB1cells were also located (Figure 1). Such co-localization at the same depth is a necessary condition for creating contacts; however, the overlap of bipolar axon terminals within ganglion dendritic areas in planar coordinates is required. In fact nearly all dendrites of PG-1 and some of the dendrites of four other parasol cells PG-2 to -5 co-stratified with the axon terminals of the 20 bipolar cells analyzed (Figure 8C). In addition, this was observed in the dendrites of cells MG-1 to -15. For example, the dendrites of cell MG-3 overlapped with the axon terminals of cells FMB-1, -2, and -3 (Figure 8D) and of cells DB1-1, -2, and -3 (Figure 8E). However, to identify real synapses we have to observe them at a higher resolution as described below.

**Figure 9:**
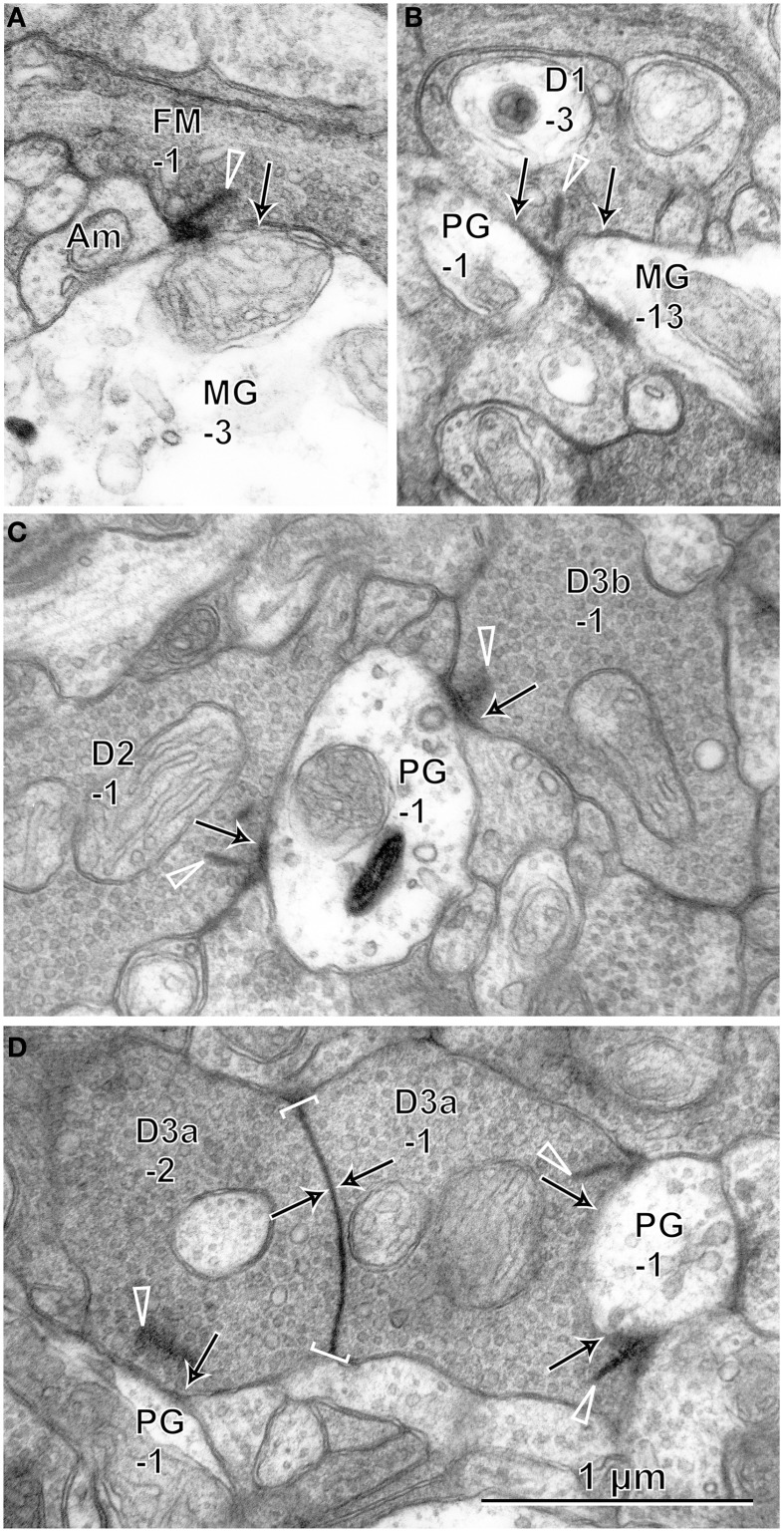
**Electron micrographs of synaptic contacts (arrows) of OFF bipolar cells with midget and parasol ganglion cells**. **(A)** The axon of cell FMB-1forms a ribbon (arrow head) synapse with the dendrite of cell MG-3. **(B)** Cell DB1-3 projects ribbon synapses to the dendrites of two cells MG-13 and PG-1. **(C)** Cells DB2-1 and DB3b-1 project ribbon synapses to the same dendrite of cell PG-1. **(D)** Cells DB3a-1 and DB3a-2 are coupled by a gap junction (arrows within brackets) and project ribbon synapses to the dendrites of cell PG-1. Bipolar letterings are abbreviated without “B.”

On electron micrographs of IPL sublayers 1 and 2 (Figure 9), we observed ribbon synapses of FMB and DB cells directed to parasol and midget ganglion cells. FMB axonal ribbon synapses were directed almost always to midget ganglion cells and only in a few cases to parasol ganglion cells. In particular, the axon tail of an FMB cell had ribbon synaptic contacts with the primary dendrite of a midget ganglion cell (Jusuf et al., 2006). DB1 synapses were more frequently directed to midget ganglion cells than to parasol ganglion cells. In contrast, DB2, 3a, and 3b synapses were normally directed to parasol ganglion cells rather than to midget ganglion cells (Jacoby et al., 2000). Adjacent DB3a axon arbors frequently exhibited homocellular (3a-3a) gap junctions (Jacoby and Marshak, 2000).

**Figure 10:**
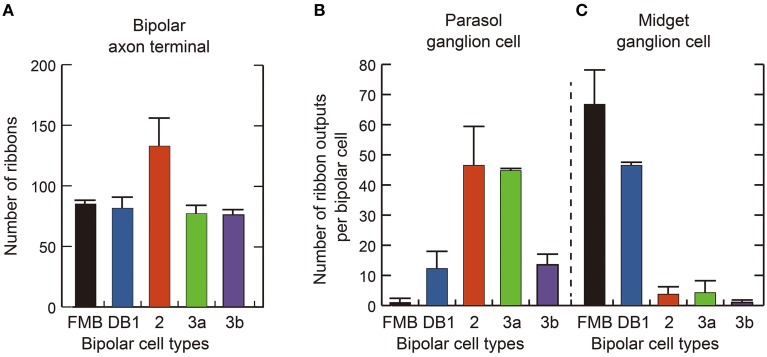
**Quantification of OFF bipolar ribbon synapses with parasol and midget ganglion cells**. **(A)** Mean number of synaptic ribbons in the axon terminals of each type of OFF bipolar cell. **(B,C)** Mean number of ribbon synaptic contacts with parasol **(B)** and midget **(C)** ganglion cells. Data are mean ± SD of four cells of each type.

Quantitative data of the bipolar-ganglion synapses is shown in Table 3 and Figure 10. DB2 had the largest number (133) of ribbons at their axon terminals, whereas FMB, DB2, 3a, and 3b cells all had similar numbers ranging from 76 to 85 (Figure 10A). Each axonal ribbon faced one to three postsynaptic processes, most frequently two processes, one to the ganglion cell and the other to the amacrine cell or both to the amacrine cell. The parasol ganglion dendritic area was usually wider than the DB axon arbor, so a given DB cell formed output synapses predominantly with only one parasol ganglion cell and less often to one or two other parasol ganglion cells. The number of synapses directed to parasol ganglion cells per DB3a or DB2 cell was 45-47 and that per DB1 or DB3b cell was 13-14. Each FMB cell formed only one synapse with a parasol ganglion cell (Figure 10B). In contrast, each FMB cell formed 67 synapses on average with only one midget ganglion cell or less often with two midget ganglion cells, one principal and the other subsidiary. The number of synapses directed to midget ganglion cells per DB1 cell was 47, that per DB2 or 3a cell was four, and that per DB3b cell was only one (Figure 10C). Thus, every OFF bipolar cell type formed synapses with both parasol and midget ganglion cells in greatly varying proportions. The number of output synapses from each OFF bipolar cell to other types of ganglion cells (not yet classified) appeared to be less than five in most cases.

**Table 3:** **Ribbon synaptic contacts of OFF bipolar cell types with parasol and midget ganglion cells**.

**(A) BC output**						**(B) MG input**	
**Bipolar cell type**	**Number of axonal ribbons**	**Outputs of a BC to PG cells**		**Outputs of a BC to MG cells**		**Inputs from BCs to an MG cell**	
		**Mean ± SD**	**%**	**Mean ± SD**	**%**	**Mean ± SD**	**%**
FMB	85.0 ± 2.9	1.0 ± 1.4	1	66.8 ± 11.4	79	166.6 ± 20.1	79.7
DB1	81.0 ± 8.5	12.5 ± 5.8	15	46.8 ± 1.5	58	33.7 ± 7.6	16.1
DB2	132.8 ± 23.2	46.8 ± 13.4	35	3.8 ± 2.5	3	6.0 ± 4.0	2.9
DB3a	77.0 ± 7.0	44.8 ± 0.5	58	4.3 ± 4.0	10	1.7 ± 2.1	0.8
DB3b	75.8 ± 3.9	13.5 ± 3.5	18	1.0 ± 0.8	7	1.0 ± 1.0	0.5
			Total inputs to an MG cell by all five cell types			209.0 ± 7.0	100

(A) The ratio of outputs to axonal ribbons for each BC type is expressed in %. n = 4 for the number of bipolar cells, (B) Partial contributions of different BC types to MG input are expressed in %. n = 3 for the number of MG cells.

BC, bipolar cell; PG, Parasol ganglion; MG, midget ganglion.

### Cone sampling strengths of midget ganglion cells

Three OFF midget ganglion cells, MG-3, -4, and -6, were located at the center of our examination area. Therefore, almost all cones that converged onto these midget ganglion cells were traced back via FMB and DB parallel pathways. Each midget ganglion cell connected with four FMB and 11-12 DB cells. The convergence of several such FMB cells onto a midget ganglion cell in this mid-peripheral retina is quite different from that in the foveal retina where one FMB cell connects to one midget ganglion cell (Kolb and Dekorver, 1991; Klug et al., 2003).

In the case of midget ganglion cell MG-3 (Figures 8D,E), it received ribbon synaptic contacts from type FMB (52, 21, and 76 contacts with FMB-1, -2, and -3 respectively) as the first most dominant input, from type DB1 (26, 11, and 2 contacts with DB1-1, -2, and -3 respectively) as the second, and from other types DB2, 3a, and 3b as the minor input (not shown). On the average of three cells MG-3, -4, and -6, a total of 209 synaptic inputs were provided by both FMB and DB cells to a midget ganglion cell (Table 3, right column). Such total synaptic inputs have not yet been available for a parasol ganglion cell in which around 100 bipolar cells are involved.

**Figure 11:**
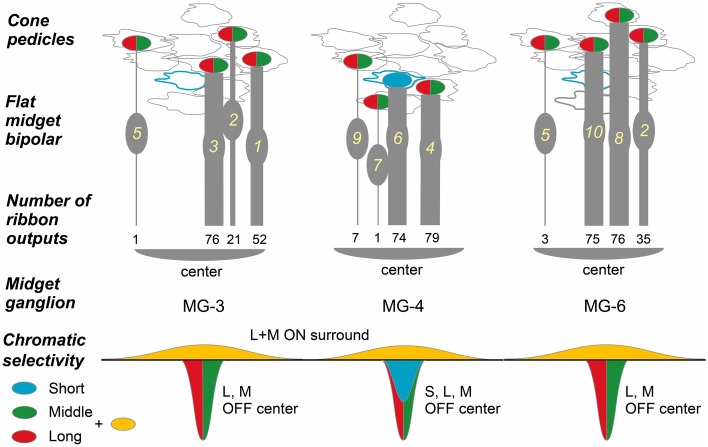
**Convergence of cones via FMB cells onto midget ganglion cells**. Four cones converge onto each of three midget ganglion cells MG-3, -4, and -6 via four FMB respective cells. Ellipses colored in red and green: L- or M-cone pedicle; ellipse colored in blue: S-cone pedicle. The numbers of ribbon synapses of FMB cells (1~10 in yellow) are provided in numerical figures and approximately expressed with the thickness of bipolar bars.

A midget ganglion cell was predominantly provided with cone signals by two of the four FMB cells (Figure 11). For example, MG-3 predominantly, moderately, and negligibly connected to FMB-1 and -3 (52 and 76 contacts respectively), FMB-2 (21 contacts), and FMB-5 (1 contact), respectively. The same FMB-2 output was shared with MG-6. Thus, the output of FMB-2 split into two halves to MG-3 and -6 (21 and 35 contacts, respectively). Particularly, MG-4 connected to FMB-4 and -6 predominantly (79 and 74 contacts respectively), and in turn FMB-4 received synaptic input from an M/L-cone (CP-20) and FMB-6 received from an S-cone (CP-26). From the chromaticity point of view, the MG-4 dendritic area may collect approximately half S-cone and half M- or L-cone signals.

**Figure 12:**
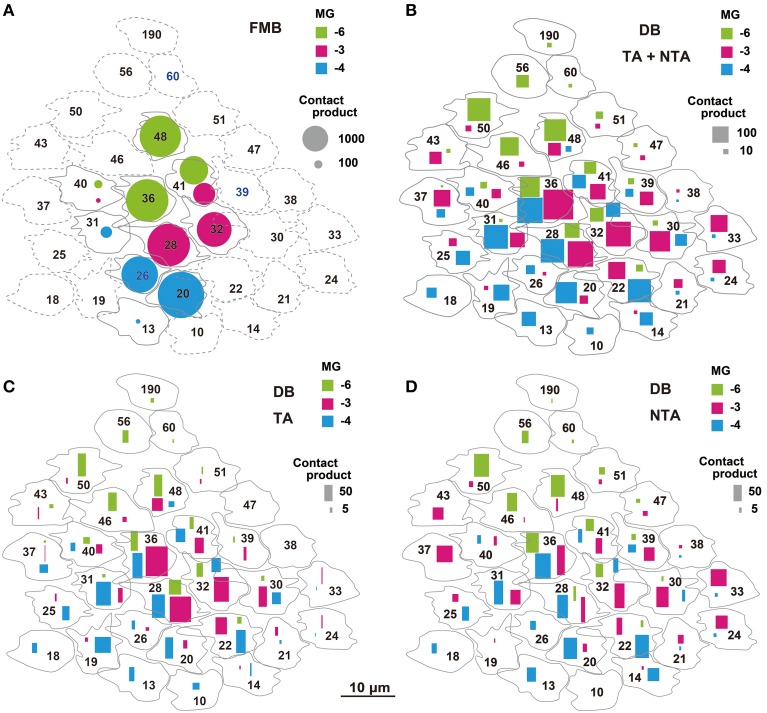
**Narrow and wide convergent cone fields of OFF midget ganglion cells estimated by the products of cone-bipolar and bipolar-ganglion contact numbers**. The product value is proportionally expressed as the area of each mark. **(A)** Narrow cone fields converging onto three adjacent OFF midget ganglion cells (MG-3 in red, MG-4 in blue, and MG-6 in green), each formed by the connectivity of two or three FMB cells. **(B)** Wide cone fields converging onto cells MG-3, MG-4, and MG-6, each formed by the connectivity of both TA and NTA contacts of all DB types. **(C,D)** Contributions of TA **(C)** and NTA **(D)** basal contacts from all types of DB cells to the wide cone field converging onto cells MG-3, MG-4, and MG-6.

We adopted the product of the number of cone-bipolar contacts and the number of bipolar- midget ganglion contacts as a measure of synaptic connectivity strength in each pathway. The total (P_g_) of the contact number products was about four-fold greater for FMB (5818 ± 691, *n* = 3) than DB cells (1552 ± 469, *n* = 3) pathways, but the number of convergent cones was about five-fold greater in DB (22 ± 3) than FMB (4.0 ± 0.0) pathways as detailed below (Figure 12).

The total number of FMB contacts per midget ganglion cell ranged from 150 to 189, 167 on average (Table 3). The products of the number of cone-FMB contacts (all kinds: TA + NTA) and the number of FMB-MG contacts in each pathway are pictorially displayed in Figure 12A. The largest product values per cone were 2736 for MG-3 with CP28, 3298 for MG-4 with CP20, and 2700 for MG-6 with CP36. The sum of the first and second largest products accounted for 86% of the total for MG-3, 96% for MG-4, and 80% for MG-6. Thus, more than 80% of the FMB-mediated cone input to these midget ganglion cells was provided by two cones.

Each of these midget ganglion cells connected to 3-5 DB1 cells via 25-39 contacts, 2-3 DB2 cells via 2-10 contacts, one DB3a cell via 1-4 contacts, and to one DB3b cell via 0-2 contacts. The total number of DB contacts per midget ganglion cell ranged from 30 to 52, 42 on average (Table 3). To estimate the DB-mediated cone input to midget ganglion cells, we first obtained the total number of basal contacts (TA + NTA). We then obtained the product of cone–DB and DB–MG synaptic junctions and displayed the cone fields of midget ganglion cells via all DB types (Figure 12B). The numbers of convergent cones via all DB types were 25 for MG-3, 23 for MG-4, and 19 for MG-6. The sum of the first to tenth largest products was 80% of the total for MG-3, 82% for MG-4, and 93% for MG-6. Thus, more than 80% of the DB-mediated cone input to these midget ganglion cells was collected from 10 cones.

The cone sampling via all DB types of midget ganglion cells was divided into two components; that mediated by TA basal contacts (Figure 12C) and that from (middle and marginal) NTA basal contacts (Figure 12D). Both TA and NTA pathways started at nearly the same group of cones. DB1 and 3a cells were mostly executed by the TA types of basal synapses, whereas DB2 and 3b cells by the NTA types (Table 1). More than half the ribbon synapses of DB1 axons were directed to midget ganglion cells, so DB1 cells were the dominant source of TA-mediated input to midget ganglion cells, followed by DB3a cells. In contrast, DB2 cells mainly provided input from NTA basal synapses. DB3b cells formed few ribbon synapse contacts with midget ganglion cells (Table 3) but they had relatively numerous basal contacts with cone pedicles (Table 1). Thus, DB3b cells still contributed cone input to midget ganglion cells, although to the lowest degree.

## Discussion

Cluster analysis of four parameters of basal contacts resulted in five distinct groups. Both the tiling and clustering properties verified the classification of the five types of OFF bipolar cells and demonstrated the distributions of the three positional classes of basal contacts (TA, middle NTA, and marginal NTA) to be OFF bipolar cell type-specific. All OFF bipolar cell types connected with greater or lesser frequency to both OFF parasol and midget ganglion cells. FMB and DB1 cells predominantly contributed to the bipolar inputs of OFF midget ganglion cells, whereas DB2 and DB3a predominantly, and DB3b moderately, contributed to the bipolar inputs of OFF parasol ganglion cells. The cone sampling routes of a midget ganglion cell consisted of two substructures: the narrow (mainly 2-3 cones) FMB pathway and the wide (mainly 10 cones) DB pathway, where connection strength is four-fold greater in the FMB than DB pathway. There was a midget ganglion cell that collected synaptic input half from an S-cone-connected FMB cell and half from an M/L-cone-connected FMB cell.

### Framework for diffusion and uptake of spillover glutamate in the intercellular cleft

In the central retina, where cone pedicles are packed in narrow space with no basal extension, marginal NTA contacts were rarely observed (Calkins et al., 1996; Calkins and Sterling, 2007). The distinction between the marginal and middle NTA contacts is thought to occur in the peripheral retina where cone pedicles can extend marginally. Furthermore, the scatter plot of the middle and marginal NTA contacts indicated a tendency for these contacts to be concomitantly expressed (Figure 7B). When Boycott and Hopkins (1993) originally defined TA and NTA basal synapses, they mentioned no distinction of two classes of NTA. However, it is highly possible that their NTA basal synapses contain both middle and marginal classes (Boycott and Hopkins, 1993; Hopkins and Boycott, 1995, 1996, 1997).

The data of TA and NTA composition in this study is thought to be consistent with the data by Hopkins and Boycott (1997) and Boycott and Hopkins (1993) as shown in Table 1. Here only four type FMB, DB1, 2, and 3 are applicable for comparison because DB3b are recently-discovered type (Puthussery et al., 2013; Tsukamoto and Omi, 2013, 2014). The previous DB3 is highly likely to correspond to DB3a. According to Hopkins and Boycott (1997), the composition of TA contacts decreases in the rank order of FMB, DB3, DB1, and DB2 in macaque retina. This is exactly the same as the rank order in the present study. The percentage of DB2 is 50% in their study, which appears to be very high, compared with 30% in our study. However, the same value is reported to be 43% in velvet monkey retina (Boycott and Hopkins, 1993), which is lesser than the 50% level. If we take the small sample size into consideration, both data are thought to be similar.

In our examination area (15°), the cone pedicles often extended the marginal edges or the filopodial processes underneath the bases of neighboring cone pedicles. The nearest ribbon synapse of such a marginal NTA basal contact was frequently found not at the contacting pedicle but at the neighboring pedicle (Figures 3E,F). In these cases, it is highly possible that the transmitter is provided mainly by spillover from the neighboring pedicle. For example, cell FMB-6 had a marginal NTA contact with CP20, where the FMB-6 dendrite may receive more spillover glutamate from CP26 than CP20 because it was closer to the ribbon synapses of CP26 than CP20 (Figure 3G). In general, marginal NTA basal contacts are thought to receive spillover glutamate in varying proportions from two pedicles adjacent to each other.

Glutamate spillover is thought to play an important role in signaling at the cone synapse. Early studies in lower vertebrates (Sarantis et al., 1988; Tachibana and Kaneko, 1988; Picaud et al., 1995) showed that cone photoreceptors respond to the glutamate released by their own ribbon synapses through the activation of an anion conductance that is intrinsic to an excitatory amino acid transporter. Recently, Szmajda and DeVries (2011) showed that a similar feedback current is present in ground squirrel cones and that the burst of glutamate released at light-off can both saturate the transporters on the releasing cone and diffuse to and activate transporters on neighboring cones. Given that glutamate can diffuse extensively at the cone synapse following ribbon-mediated release, our observations are consistent with the idea that diffusional filtering (Rao-Mirotznik et al., 1998; DeVries et al., 2006) might shape the temporal responses of the different types of primate OFF bipolar cells based on the average distance of their contacts from ribbon sites.

OFF bipolar cells may express different types of kainate or AMPA receptors on their dendrites. In macaque retinas, Haverkamp et al. (2001a) showed via immunocytochemistry that kainate receptors are expressed to a greater extent by OFF bipolar cells than by horizontal cells and that GluR5 (GluK1) and GluR6/7 (GluK2/3) tend to be expressed at NTA and TA contacts, respectively. They also demonstrated that the AMPA receptor subunits GluR1–4 (GluA1–4) are expressed to a lesser extent by OFF bipolar cells than by horizontal cells; however, within the OFF bipolar cells, these receptors are more extensively expressed at TA basal contacts (49%) than at NTA basal contacts (17%) (Haverkamp et al., 2001b). To further study GluK1 expression in OFF bipolar cells, Puthussery et al. (2014) performed double immunolabeling using antibodies against GluK1 together with markers for different bipolar cell types. They found that DB2 and DB3b cells express GluK1 but FMB, DB1, and DB3a cells do not. By combining this with the above-mentioned finding that GluK1 tends to be expressed at NTA contacts, one may predict that DB2 and DB3b cells are accompanied with NTA contacts. In fact, our results showed that approximately three-fourths of the basal synapses on DB2 and DB3b cell dendrites are NTA contacts (Table 1, Figure 6C). Thus, some extent of heterogeneity in ionotropic glutamate receptor expression has been clarified, but it is still unknown which types of OFF bipolar cells of the macaque retina express which types of receptor subunits.

Results from the ground squirrel (DeVries, 2000; DeVries et al., 2006) led to the idea of a dichotomy between transient bipolar cells, such as CB2 cells that have a high proportion of TA contacts and use AMPA-type receptors with fast recovery from desensitization, and sustained cells, such as CB3 cells that have a high proportion of NTA contacts and use kainate-type receptors with slow recovery. However, AMPA receptors do not appear to play a major role in mediating transmission at the cone to OFF bipolar cell synapse in the macaque (Puthussery et al., 2014). Rather, an analogous role might be played by the two kinetically distinct types of kainate receptors that have been described at cone synapses in ground squirrels (DeVries et al., 2006; Lindstrom et al., 2014) and mice (Borghuis et al., 2014).

Puthussery et al. (2014) found that the puffer application of glutamate onto the kainate receptors of macaque OFF bipolar cells evoked transient responses in DB2 and DB3b cells but sustained responses in FMB, DB1, and DB3a cells. Our work shows that the cells with more transient glutamate responses (DB2 and DB3b cells) have a high proportion of NTA contacts, whereas those with sustained glutamate responses (FMB, DB1, and DB3a cells) have a high proportion of TA contacts (Table 1, Figure 6C). The association between sustained glutamate response and predominant TA location vs. transient response and predominant NTA location is the opposite of that suggested by the ground squirrel results. However, there are at least three caveats concerning the results obtained in primates. First, glutamate applied via a puffer pipette may initially arrive at NTA contacts and then at TA contacts and may also be too slow to adequately resolve the temporal properties of the AMPA/kainate receptors. Second, the effects of glutamate transporters on the glutamate concentration may differ between endogenous release and exogenous application but are not adequately assessed. Third, a list of the subunit compositions with the auxiliary subunits of the receptors for all OFF bipolar types has not been exactly determined, and the recovery time course of each receptor following a desensitizing pulse of glutamate is not known. Thus, how the predominance of TA or NTA contacts is correlated with receptor subunit type and electrophysiological events *in situ* remains to be fully elucidated in primates.

### Scarcity of basal contacts in S-cone pedicles

Although every type of OFF bipolar cell tiled the same field of cone pedicles, DB cell dendrites tended to evade S cone pedicles to varying degrees (Figure 6E). Lee and Grünert (2007) clarified the overall tendency in DB cell avoidance of S cones in macaque and marmoset retinas. In addition to the difference of S cones from M/L cones in connectivity with all types of DB cells, we found that DB1 and DB3a more selectively escaped from S cones. In mice, type 1 bipolar cells avoided S cones (Breuninger et al., 2011). Because the mouse type 1 corresponds to macaque type DB1 (Tsukamoto and Omi, 2014), this selective avoidance of S cones may be pervasive across mammals. In contrast, Breuninger et al. (2011) found that type 3a cells in mice, corresponding to DB3a cells, indiscriminately contacted both S and M cones. These findings may implicate that DB1 and DB3a have different roles in chromaticity processing in retinal circuits.

### Ribbon synaptic contacts at bipolar-ganglion junctions

Whether the recently discovered bipolar cell type DB3b has synaptic connections with parasol ganglion cells has not been directly demonstrated (Puthussery et al., 2013). In the present study, synaptic contacts of DB3b cells with parasol ganglion cells were observed, although the number of contacts was 3–4-fold lower compared with DB2 and DB3a cells. Synapses between DB3b and midget ganglion cells were rare, so the DB3b type is thought to belong to the parasol ganglion/magnocellular pathway. Furthermore, DB3b cells are known to directly connect with rod terminals (Tsukamoto and Omi, 2013, 2014), which may contribute to the transmission of fast rod signals to parasol ganglion cells under mesopic light conditions. DB3b cells may facilitate the smooth mode transition from rod (scotopic) to cone (photopic) vision.

It was surprising that DB1 cells provided about four-fold greater input to midget than parasol ganglion cells, suggesting that the midget ganglion/parvocellular pathway is not solely driven by FMB cells but is supplemented by DB1 cells. Indeed, the number of DB1-midget ganglion synapses was about 70% that of FMB-midget ganglion synapses. FMB and DB1 cells contribute predominantly to the midget ganglion pathway and DB2 and DB3a cells to the parasol ganglion pathway but certain differences in spatial characteristics are noted; FMB is narrower than DB1 in the dendritic area and DB2 is narrower than DB3a.

### Narrow and wide receptive cone fields converging onto midget ganglion cells

Electrophysiological findings that the receptive fields of parvocellular cells were almost as wide as those of the magnocellular cells (Derrington and Lennie, 1984; Spear et al., 1994; Levitt et al., 2001) may be partly explained by the wide cone sampling area evaluated by the contact number products in this study. Although, the dendritic area diameter of midget ganglion cells was about one fifth that of parasol ganglion cells, the cone sampling area diameter of midget ganglion cells via DB cells was almost equal to the dendritic area diameter of parasol ganglion cells (Figures 8, 12).

The receptive fields of macaque retinal ganglion cells were analyzed with the spike-triggered average method at the resolution of cones (Field et al., 2010). The number of effective cones converging onto the receptive field center of a midget ganglion cell ranged from 12 to 19, which were slightly greater than approximately 10 in the present study. Because they recorded at 6.8 mm eccentricity, the midget ganglion dendritic area is thought to be slightly greater than that in our case at 3 mm eccentricity. Concerning the underling anatomical connectivity in the “black box” of their cone sampling routes of a midget ganglion cell, our study suggests that a few central cones with strong connectivity may be conveyed by FMB cells and many other surrounding cones may be conveyed by DB cells.

The connection strength of the narrow field via FMB cells was estimated to be four-fold stronger than the wide field via DB cells, so the wide field responses might have been easily masked by the narrow field responses in electrophysiological studies, probably depending on the adaptation levels. The highest spatial resolution may be conferred by the narrow and strong FMB pathway.

## Author contributions

The authors had full access to all the data in the study and take full responsibility for the integrity and the accuracy of the data analysis. YT designed this study, took micrographs, acquired data, interpreted results, and wrote the manuscript. NO took micrographs, acquired data, and checked the manuscript.

### Conflict of interest statement

The authors declare that the research was conducted in the absence of any commercial or financial relationships that could be construed as a potential conflict of interest.
